# Transcriptome response of proliferating muscle satellite cells to thermal challenge in commercial turkey

**DOI:** 10.3389/fphys.2022.970243

**Published:** 2022-08-25

**Authors:** Kent M. Reed, Kristelle M. Mendoza, Gale M. Strasburg, Sandra G. Velleman

**Affiliations:** ^1^ Department of Veterinary and Biomedical Sciences, University of Minnesota, Falcon Heights, MN, United States; ^2^ Department of Food Science and Human Nutrition, Michigan State University, East Lansing, MI, United States; ^3^ Department of Animal Sciences, The Ohio State University/Ohio Agricultural Research and Development Center, Wooster, OH, United States

**Keywords:** satellite cell, proliferation, skeletal muscle, growth selection, Turkey

## Abstract

Thermal stress poses a threat to agricultural systems through increased risk to animal growth, health, and production. Exposure of poultry, especially hatchlings, to extreme temperatures can seriously affect muscle development and thus compromise subsequent meat quality. This study was designed to characterize transcriptional changes induced in turkey muscle satellite cells (SCs) cultured from commercial birds under thermal challenge to determine the applicability of previous results obtained for select research lines. Satellite cells isolated from the pectoralis major muscle of 1-week old commercial fast-growing birds (Nicholas turkey, NCT) and from a slower-growing research line (RBC2) were proliferated in culture at 38°C or 43°C for 72 h. RNAseq analysis found statistically significant differences in gene expression among treatments and between turkey lines with a greater number of genes altered in the NCT SCs suggesting early myogenesis. Pathway analysis identified cell signaling and regulation of Ca^2+^ as important responses. Expression of the intercellular signaling Wnt genes, particularly *Wnt5a* and *7a* was significantly altered by temperature with differential response between lines. The peripheral calcium channel *RYR3* gene was among the genes most highly upregulated by heat stress. Increased expression of *RYR3* would likely result in higher resting cytosolic calcium levels and increased overall gene transcription. Although responses in the calcium signaling pathway were similar among the RBC2 and NCT lines, the magnitude of expression changes was greater in the commercially selected birds. These results provide evidence into how SC activity, cellular fate, and ultimately muscle development are altered by heat stress and commercial selection.

## Introduction

A major agricultural concern is a decline in food and feed production in geographic regions experiencing adverse effects of climate change. Temperature and humidity extremes are especially detrimental to livestock, including poultry, with hatchlings and pre-market birds being particularly vulnerable at this critical stage of development. Contemporary commercial poultry have been genetically selected over several decades for increased growth, feed conversion, and breast (pectoralis major) muscle yield, resulting in larger, faster growing birds with altered muscle morphology, especially at the microscopic level. These changes result in muscle that generates greater metabolic heat (Konarzewski et al., 2000) coupled with reduced capillary supply (relative to mass) that increases the concentration of anaerobic respiration products (Hoving-Bolink et al., 2000; Velleman et al., 2003; Joiner et al., 2014). As a result, faster-growing commercial birds have lower heat tolerance and suffer increased muscle defects due to heat stress (Wilson et al., 1990; Berrong and Washburn, 1998; Velleman et al., 2003; Chiang et al., 2008). The potential economic losses due to reduced production and meat quality are of particular concern to poultry producers.

Growth and development of muscle are dependent on the activity of multipotent stem cells (satellite cells, SCs) that have the ability to follow myogenic, osteogenic, or adipogenic cellular pathways (Asakura et al., 2001; Powell et al., 2016). The number of muscle myofibers is fixed at hatch (Smith, 1963) and post-hatch growth is the result of active proliferation of progenitor SCs followed by fusion to form myofibers (Moss and Leblond, 1971). During the first week of life of the post-hatch period, SCs have high levels of mitotic activity needed for hypertrophic growth; and after this period the cells become quiescent until they are needed for repair (Mozdziak et al., 1994; Halevy et al., 2001; Bi and Kuang, 2012). In response to heat stress, avian SCs show accelerated proliferation and differentiation (Halevy et al., 2001) with cells being more sensitive to environmental temperatures during proliferation (Xu et al., 2021a). Thus, post-hatch thermal stress can potentially change myogenic potential by altering SC heterogeneity. These observations are of particular importance because of the nature of the poultry industry. Following hatch birds are transported to farms, frequently over long distances, and are potentially exposed to temperature extremes. Because they have immature thermoregulatory systems, their body temperatures, especially near the body surface, may be significantly elevated thereby triggering adverse muscle development when SCs are highly active.

In a previous study (Reed et al., 2017a), we contrasted effects of thermal challenge on proliferating turkey skeletal muscle SC transcriptomes from select research lines: RBC2 (Randombred control line 2) and F line (selected for only 16 weeks body weight from the RBC2 line) (Nestor, 1977; 1984). The SCs used in that study (isolated from 7-week old male birds) showed a general increase in expression of genes related to muscle system development and differentiation with heat-treatment. The activation and proliferation of SCs is controlled by signaling molecules and myogenic regulatory transcription factors such as MYOD1 (Myogenic Differentiation 1) and interacting genes. These genes were significantly upregulated in SCs of the growth selected line with heat treatment (Clark et al., 2016; Reed et al., 2017a). The “Wingless-related integration site” Wnt gene (Wnt7a) important in the induction of satellite cells, is implicated in several developmental processes, including regulation of cell fate and patterning during embryogenesis (Eubelen et al., 2018). This gene was also significantly upregulated indicating potential differential control of SC proliferation with growth selection. Thus, these studies identified important genes and pathways of potential significance to heat response in commercial turkeys.

The present study was designed to determine the applicability of previous results obtained for select research lines to that of commercial birds by examining transcriptional changes induced in SCs under thermal challenge. Here we examine gene expression in SCs derived from 1-week old commercial fast-growing birds and from the slower-growing RBC2 line. Importantly, this corresponds to the period of maximal mitotic activity and period of SC response to external stimuli like temperature. We hypothesize that satellite cell activity, cellular fate, and muscle development are altered by age of the SCs and heat stress. We contrast the results from SCs of commercial birds with that of the research lines used in previous studies. Understanding the effects of heat stress on satellite cell-mediated mechanisms will allow development of effective breeding, nutritional, and management strategies to enhance production of consistent, high-quality muscle food products and minimize impact of climatic stressors.

## Methods

### Turkey myogenic satellite cells

Satellite cells were previously isolated from the p. major muscle of 1-week-old male Nicholas Commercial (NCT) turkeys and RBC2 turkeys as described by Velleman et al. (2000) and stored in liquid nitrogen until use. The NCT turkeys are modern commercial meat-type turkeys selected for increased growth rate and breast muscle yield and were obtained from Nicholas Turkeys (Lewisburg, WV). The RBC2 turkeys were initiated in 1966 by crossing two commercial large white turkey strains representing the genetics of the commercial turkeys at the time (Nestor et al., 1969). The RBC2 line was then maintained at the Poultry Research Center of the Ohio Agricultural Research Development Center/The Ohio State University, Wooster, OH without conscious selection for any trait. Only SCs from males were used to avoid any sex effects (Velleman et al., 2000).

Turkey p. major SCs were cultured as described in Reed et al. (2017a). Briefly, cells were plated and incubated at 38°C for 24 h in plating medium consisting of Dulbecco’s Modified Eagle’s Medium (DMEM, Sigma Aldrich, St. Louis, MO), 10% chicken serum (Gemini BioProducts, West Sacramento, CA), 5% horse serum (Gemini BioProducts), 1% antibiotics-antimycotics, and 0.1% gentamicin. After 24 h the cells were fed growth medium and cultured at an experimental temperature (38° or 43°C) for 72 h. The control temperature of 38°C is approximately that measured in newly hatched poults (38.0–38.5°C, Strasburg, unpublished data). The heat stress temperature of 43°C exceeds the approximate body temperature of mature turkeys (41.5°C) and has been shown to have significant effects on SC proliferation (Clark et al., 2016; Xu et al., 2021a). Growth medium was changed every 24 h during the 72 h treatment. At harvest, cell medium was removed and the cells were collected into RNAzol RT (Sigma-Aldrich) and held at −80°C until RNA isolation.

### RNA isolation and sequencing

Total RNA from each sample was isolated by RNAzol RT extraction, DNase-treated (Turbo DNA-freeTM Kit, Ambion, Inc.), and stored at −80°C. Initial RNA concentration and quality were assessed by spectrophotometry (Nanodrop 1000), and samples were submitted for library preparation and sequencing at the University of Minnesota Genomics Center. Each sample was further quantified by RiboGreen Assay (Invitrogen Corp.) and RNA integrity confirmed on the 2100 Bioanalyzer (Agilent Technologies). For each treatment group, replicate samples were prepped for sequencing (*n* = 2 biological replicates per treatment group). RNA Integrity Numbers (RIN) ranged between 5.2 and 9.0. Indexed libraries were constructed with 1 μg of total RNA/sample with the TruSeq Stranded mRNA Sample Preparation Kit (Illumina, Inc.) and size selected for approximately 200 bp inserts. Libraries were multiplexed and sequenced on the NovaSeq SP platform using v1.5 chemistry (Illumina, Inc.) to produce 51-bp paired-end reads.

### RNAseq data analyses

Sequence adapters were removed and low quality bases were trimmed using Trimmomatic (Bolger et al., 2014) enabled with the optional “−q” option; 3 bp sliding-window trimming from 3’ end requiring minimum Q30. Quality control checks on raw sequence data for each sample were performed with FastQC (Andrews, 2010). Read mapping was performed via Bowtie (v2.2.4.0) using the turkey genome (UMD 5.1, ENSEMBL Annotation 104). Read counts were normalized in CLC Genomics Workbench (CLCGWB v. 10.1, CLC Bio).

Identifiers for annotated genes were iteratively obtained from several sources. The majority were retrieved directly from the ENSEMBL annotation. For genes with empty ENSEMBL ID fields, additional gene IDs were obtained from individual searches of DAVID, Uniprot and NCBI using the Ensembl IDs. For the remaining “novel” genes, individual searches were conducted in ENSEMBL for orthologs in the chicken genome (GRCg6a). This resulted in approximately 2500 genes in the turkey gene list remaining as uncharacterized or novel protein coding genes.

Hierarchical clustering of samples (based on Euclidean sample distances with single linkage) was performed in CLCGWB using normalized reads counts. Empirical analysis of differential gene expression and ANOVA was performed in CLCGWB on original expression values (Bonferroni and FDR corrected). Principal component analysis (PCA) was performed in CLCGWB and pair-wise comparisons between treatment groups were made in the Bioconductor (3.2) R package DESeq2 (Love et al., 2014) following the standard workflow. Gene enrichment tests were performed using the PANTHER Overrepresentation Test [GO Consortium release 20150430 (Mi et al., 2013); http://geneontology.org/]. For GO analysis the reference gene list of the chicken (*Gallus gallus*) was utilized with ∼66% of the turkey loci (Annotation 105) having ID homologs as presented in Supplementary Table S1. Additional analyses were conducted through the use of Qiagen Ingenuity Pathway Analysis (Krämer et al., 2014).

## Results

### Gene expression

Total RNA isolated from SC cultures (*n* = 8) was used to construct individual barcoded libraries. Sequencing produced over 181M paired-end reads (accessioned as part of SRA BioProject PRJNA842679). The number of reads per library ranged from 20.7 to 25.7M (average 22.7M) (Table 1). After read trimming and filtering, read quality was consistently high and Q values ranged from 35.8 to 36.3. Replicate libraries produced comparable results with an average difference between replicates of 2.2M reads.

**TABLE 1:** Summary of RNAseq data for proliferation experiment. For each library the total number of raw reads, median read qualities (R1 and R2), the number of observed genes (mapped reads >1) by library and treatment group and the percentage of expressed genes (group read count >1.0) are given.

Line	Temp °C	Replicate	PE reads	Median read quality R1	Median read quality R2	Observed genes	Mean observed genes	Group observed genes	% Expressed genes
RBC2	38	A	20752520	36.2	35.8	13019	13066.5	13656	0.760
		B	25685620	36.2	35.8	13114			
	43	A	22082964	36.2	35.8	13153	13089.5	13653	0.760
		B	21440710	36.3	35.8	13026			
NCT	38	A	22171929	36.3	35.9	13217	13072.5	13657	0.760
		B	21604179	36.2	35.8	12928			
	43	A	25324202	36.2	35.8	13444	13414.0	13949	0.776
		B	22527686	36.3	35.8	13384			
Mean			22698726.25	36.24	35.81	13160.625	13160.63	13728.75	0.7640

**FIGURE 1:**
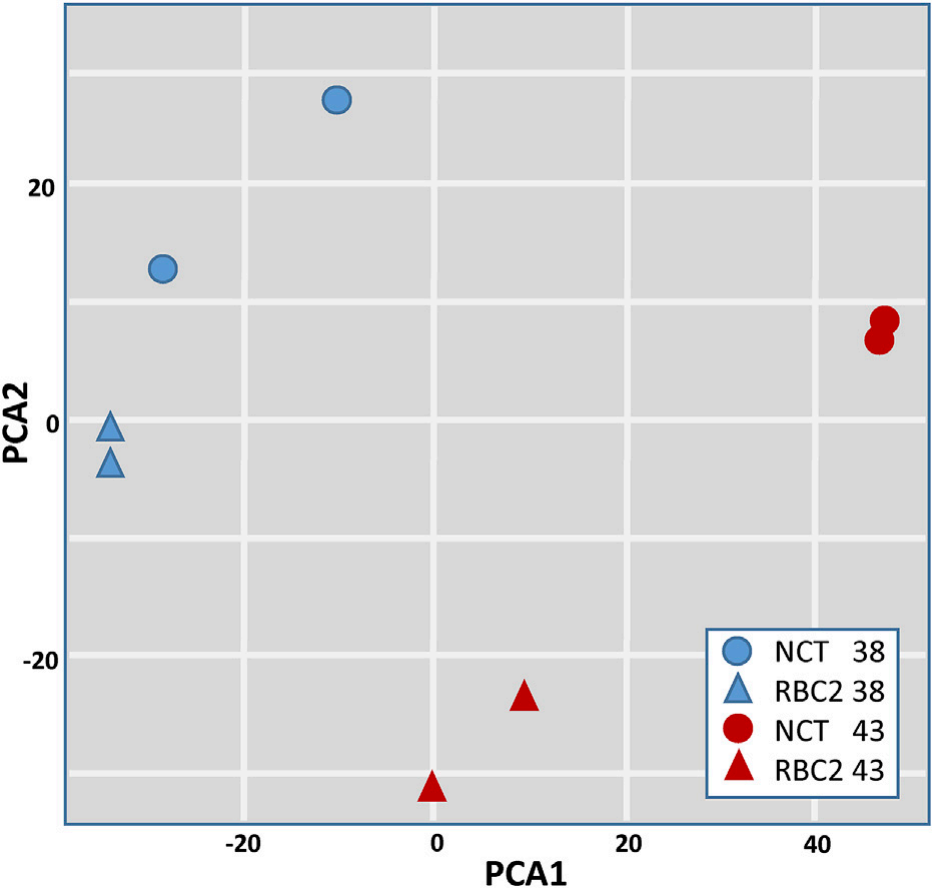
Principal component (PCA) plot of RNAseq data based on normalized read counts. Sample to sample distances (within- and between-treatments) are illustrated on the first two principal components. Samples are plotted according to treatment.

Evidence for expression (at least one mapped read in at least one treatment group) was observed for 15,118 genes and 76.4% of the ENSEMBL turkey gene set (80.3% of protein-coding genes) (Supplementary Table S1). The number of observed genes per library ranged from 12,928 to 13,444 (average 13,160.6), whereas the number of observed genes per treatment group ranged from 13,653 to 13,949 (average 13,728.7) (Table 1). Variation among treatment groups was visualized by principal component analysis (PCA) based on normalized read counts (Figure 1). Treatment groups clustered distinctly (Temp/Time) within the first two principal components irrespective of line. Replicate treatment pairs occurred as nearest neighbors within the PCA space, supporting the pooling of replicates for expression analyses.

Differences in gene expression among groups is demonstrated in the distribution of unique and shared expressed genes among treatment groups as illustrated in Table 2. On average 13,031 genes were expressed in the between-treatment group comparisons within lines (Temp effect), and 12,080 being shared. Characterization of the suite of expressed genes in the SCs derived from muscle from 1-week old birds was consistent with that observed in our previous study of cells from 7-week birds with enzymes, transcription regulators and transporter being the most represented functional class separate of structural proteins (Supplementary Table S1). Numbers of uniquely expressed genes were higher at the higher temperature (460, and 650 in the RBC2 and NCT lines, respectively). Within temperature an average of 12,972 genes were expressed between lines, with 12,140 being shared. This reflects an overall similarity in response of cultured SCs between the turkey lines. Here, the number of uniquely expressed genes were higher in the RBC2 at 38°C but higher in the NCT lines at 43°C.

**TABLE 2:** Summary of gene expression and significant differential expression (DE) in pair-wise comparisons of proliferating cells.

	Comparison	Total expressed genes	Shared genes	Unique genes in each group	FDR <0.05	|Log_2_FC| > 1.0	|Log_2_FC| > 2.0
Temp effect							
	RBC2 (43**°**C vs. 38**°**C)	12947	12044	460/443	4241	1112	354
	NCT (43**°**C vs. 38**°**C)	13115	12116	650/349	4465	2590	730
Line effect							
38°C	NCT vs. RBC2	12897	12055	410/432	627	439	159
43°C	NCT vs. RBC2	13046	12224	542/280	7393	2389	492

For each comparison of the treatment groups (Temperature: 38°C or 43°C, Line: RBC2 or NCT), the total number of expressed and uniquely expressed genes, the number of genes with significant FDR *p*-value, and the numbers of significant genes also with |Log_2_ fold change| > 1.0 and >2.0 are given. Only those genes with treatment group mean normalized read counts >1.0 are counted as expressed.

### Temperature effects

Temperature effects were examined in two pairwise, within-line comparisons: RBC2 hot-versus control (43 vs. 38°C) and NCT hot versus control (43 vs. 38°C) (Supplementary Table S2). Each of these within-line comparisons identified over 4000 significant differentially expressed (DE) genes (FDR adjusted *p*-value < 0.05, Table 2) in the SCs; a greater number of DE genes was observed in the NCT SCs (4465) compared to the RBC2 (4241). These numbers are substantially reduced when limited by fold change. For example, the number of DE genes in the RBC2 group comparison is reduced to 1,112 and 354 (|Log_2_FC| > 1.0, and >2.0, respectively).

**FIGURE 2:**
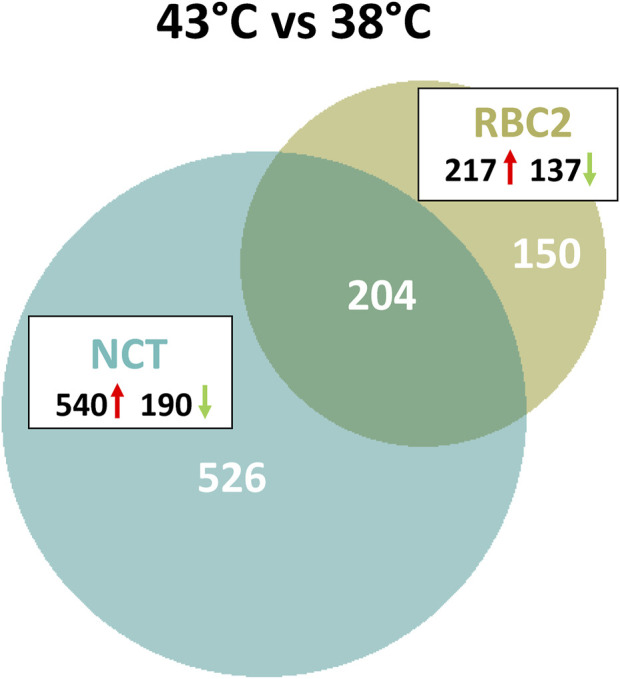
Distribution of differentially expressed genes during proliferation of cultured turkey p. major satellite cells. For each temperature comparison, the number of genes (FDR pval <0.05 and |Log_2_FC| > 2.0) shared or unique to each line (RBC2 and NCT) are indicated in the Venn diagram. Circle size is proportional to the number of genes.

**FIGURE 3:**
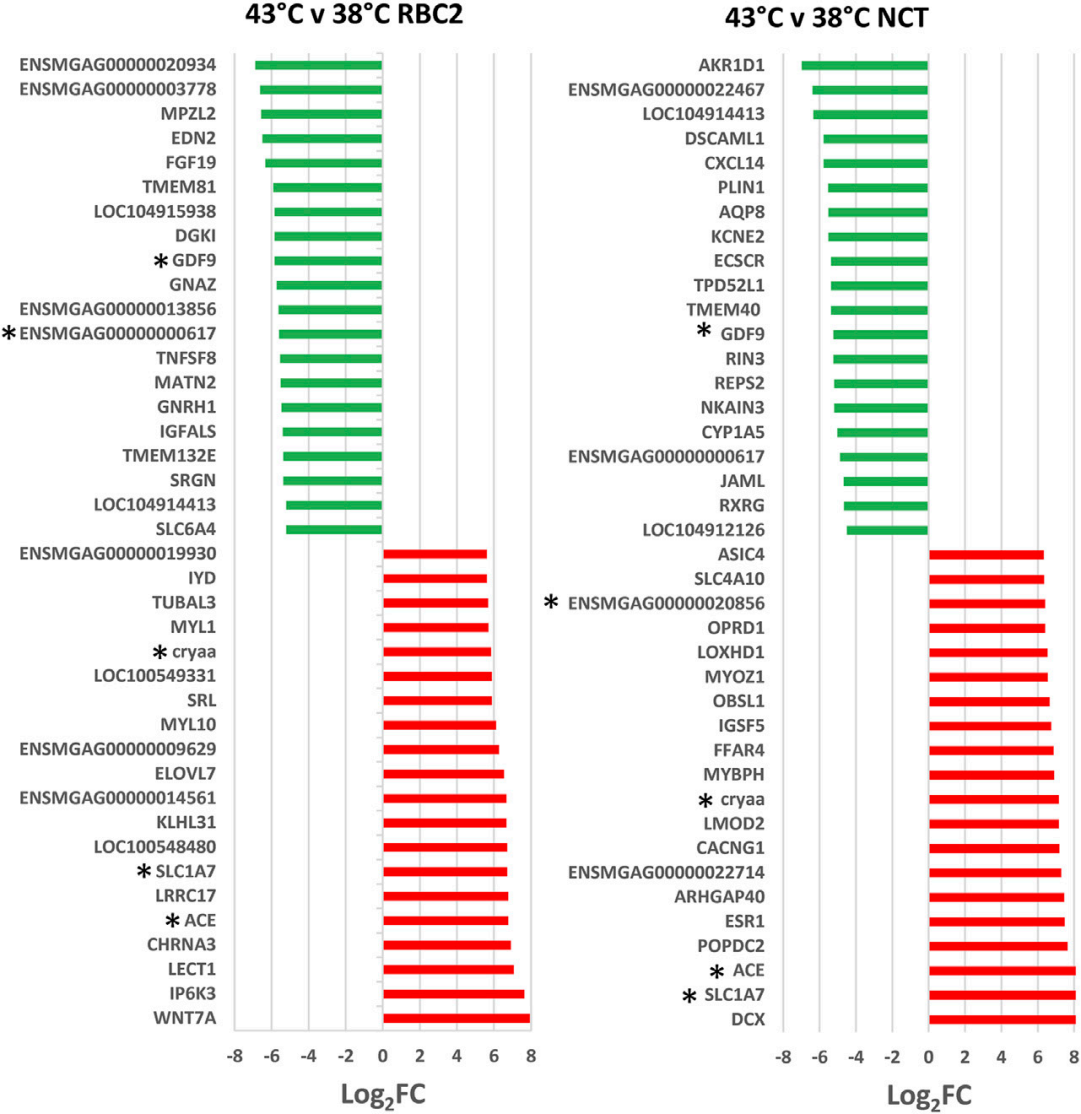
Directional change in the genes showing the highest expression differences between lines (NCT and RBC2) at 38 and 43°C. For each with-in temperature comparison the Log_2_FC for each gene is plotted (FDR pval <0.05). Genes shared between temperatures are indicated by asterisks.

As illustrated in Figure 2, a total of 880 genes were identified with significant DE (*p* < 0.05 and |Log_2_FC| > 2.0) in the comparison of SCs proliferated at 43 versus 38°C with 204 shared between the lines. Of the 354 DEGs significant in the RBC2 SCs, 217 were upregulated and 137 down regulated with heat treatment (43°C compared to 38°C) (Supplementary Table S2). Unique to the RBC2 cells were 150 DEGs altered by heat challenge. The 40 significant DEGs showing the greatest fold change are listed in Figure 3. Genes down regulated in the RBC2 line included two loci (*ENSMGAG00000003778* and *ENSMGAG00000020934*) with similarity to selectin E (*SELE*). In mammals, this protein-coding gene is expressed in cytokine-stimulated endothelial cells and is involved in inflammation by mediating adhesion of cells to the vascular lining. Also included are genes for structural proteins {*MPZL2* (myelin protein zero like 2), *ENSMGAG00000000617* (Tubulin beta chain), integral membrane proteins [*TMEM81* (transmembrane protein 81), *TMEM132E* (transmembrane protein 132E), *SLC6A4* (solute carrier family 6 member 4)], and growth factors [*FGF19* (fibroblast growth factor 19), *GDF9* (growth differentiation factor 9), *IGFALS* (insulin like growth factor binding protein acid labile subunit)]}; some of these genes have potential roles in development. Up regulated genes include *WNT7a*, a Wnt family member. Genes in this family are involved in several developmental processes including regulation of cell fate and patterning. Several genes associated with muscle function are also included: [*LOC100549331* (myosin-7-like), *MYL1* (myosin light chain 1), and *MYL10* (myosin light chain 10), and *TUBAL3* (tubulin alpha-3 chain)]. Pathway analysis (PANTHER) of the 354 RBC2 DEGs identified the inflammation mediated by chemokine and cytokine signaling pathway (P00031), Wnt signaling pathway (GO:P00057), and Angiogenesis (GO:P00005) as affected pathways. Overrepresentation test of the up- and down-regulated gene sets suggest heat treatment up regulates muscle function and down regulates endocrine response (Table 3).

**TABLE 3:** PANTHER Overrepresentation test of 354 DEGs in the RBC2 line temperature comparison (43°C vs. 38°C) after 72 h of proliferation. Shown are the three gene ontology categories with the greatest fold enrichment in each category for the up and down-regulated genes^a^.

Up regulated DEGs					
	*Gallus gallus* (18109)	Turkey DEGs (164 of 217)	Expected	Fold enrichment	*p*-value
GO biological process complete					
tonic smooth muscle contraction (GO:0014820)	10	4	0.09	44.2	4.06E-02
skeletal muscle contraction (GO:0003009)	14	5	0.13	39.4	4.00E-03
sarcomere organization (GO:0045214)	31	9	0.28	32.1	4.54E-07
GO molecular function complete					
troponin C binding (GO:0030172)	4	4	0.04	>100	1.00E−03
troponin I binding (GO:0031013)	6	4	0.05	73.61	2.96E−03
tropomyosin binding (GO:0005523)	15	6	0.14	44.17	5.44E−05
GO cellular component complete					
troponin complex (GO:0005861)	11	6	0.1	60.23	6.85E−06
striated muscle thin filament (GO:0005865)	19	8	0.17	46.49	8.57E−08
myofilament (GO:0036379)	20	8	0.18	44.17	1.19E−07

**Down regulated DEGs**					
	***Gallus gallus* (18109)**	**Turkey DEGs (81 of 137)**	**Expected**	**Fold Enrichment**	***p*-value**
GO biological process complete					
endocrine process (GO:0050886)	43	5	0.19	26.0	1.58E−02
system process (GO:0003008)	1067	17	4.77	3.6	3.17E−02
regulation of response to stimulus (GO:0048583)	2693	29	12.05	2.4	3.32E−02
GO molecular function complete					
hormone activity (GO:0005179)	100	6	0.45	13.4	1.82E−02
cytokine activity (GO:0005125)	138	8	0.62	13.0	6.55E−04
growth factor activity (GO:0008083)	119	6	0.53	11.3	4.67E−02
GO cellular component complete					
extracellular space (GO:0005615)	1221	19	5.46	3.5	1.97E−03
extracellular region (GO:0005576)	1657	20	7.41	2.7	4.26E−02
cell periphery (GO:0071944)	4222	37	18.88	2.0	1.22E−02

a Turkey DEGs were matched to the chicken reference gene list IDs for analysis in PANTHER. For each category, the number of genes in the reference list and turkey DEGs are given. *p*-values are as determined by Fisher Exact Test with Bonferroni correction.

A greater number of genes were affected by heat treatment (730 total DEGs) in the NCT SCs, with 540 being upregulated and 190 down regulated (Figure 2, Supplementary Table S2). Unique to the NCT cells were 526 DEGs altered by heat challenge. Genes showing the largest fold change (Figure 3) include a suite of diverse structural, enzymatic, physiological, and immunological functions. Muscle-associated genes such as *MYOZ1* (myozenin 1), *MYBPH* (myosin binding protein H) and *CACNG1* (calcium voltage-gated channel auxiliary subunit gamma 1) were significantly upregulated by heat treatment. However, genes with putative vascular/endothelial functions were both up-regulated [*ACE* (angiotensin I converting enzyme)] and down-regulation [*ECSCR* (endothelial cell surface expressed chemotaxis and apoptosis regulator), *KCNE2* (potassium voltage-gated channel subfamily E regulatory subunit 2), and *LOC104912126* (type-1 angiotensin II receptor-like)]. Pathway analysis (PANTHER) of the 730 NCT DEGs identified the Nicotinic acetylcholine receptor signaling (4.04 fold) and the Inflammation mediated by chemokine and Cytokine signaling (2.97 fold, *p* = 7.70E−03) as significantly enriched pathways. Overrepresentation test of the up- and down-regulated gene sets found heat treatment to up regulate muscle structural genes and down regulate regulation of multicellular organismal processes (GO:0051240) (Table 4).

**TABLE 4:** PANTHER Overrepresentation test of 730 DEGs in the NCT line temperature comparison (43°C vs. 38°C) after 72 h of proliferation. Shown are the gene ontology categories with the greatest fold enrichment in each category for the up and down-regulated genes^a^.

Up regulated DEGs					
	*Gallus gallus* (18109)	Turkey DEGs (371 of 540)	Expected	Fold enrichment	*p*-value
GO biological process complete					
troponin complex (GO:0005861)	11	5	0.23	22.19	1.45E−02
striated muscle thin filament (GO:0005865)	19	7	0.39	17.98	7.40E−04
myofilament (GO:0036379)	20	7	0.41	17.08	9.82E−04
GO molecular function complete					
troponin C binding (GO:0030172)	4	4	0.08	48.81	2.47E−02
tropomyosin binding (GO:0005523)	15	6	0.31	19.52	6.21E−03
postsynaptic neurotransmitter receptor activity (GO:0098960)	74	10	1.52	6.6	1.63E−02
GO cellular component complete					
sarcomere organization (GO:0045214)	31	11	0.64	17.32	3.14E−06
myofibril assembly (GO:0030239)	55	14	1.13	12.42	5.36E−07
striated muscle cell development (GO:0055002)	56	14	1.15	12.2	6.58E−07

Down regulated DEGs					
	***Gallus gallus* (18109)**	**Turkey DEGs (122 of 190)**	**Expected**	**Fold Enrichment**	***p*-value**
GO biological process complete					
positive regulation of multicellular organismal process (GO:0051240)	858	19	5.78	3.29	3.96E−02
regulation of multicellular organismal process (GO:0051239)	1683	29	11.34	2.56	1.50E−02
GO molecular function complete					
signaling receptor regulator activity (GO:0030545)	386	12	2.6	4.61	3.49E−02
molecular function regulator (GO:0098772)	1441	25	9.71	2.58	2.66E−02

a Turkey DEGs were matched to the chicken reference gene list IDs for analysis in PANTHER. For each category, the number of genes in the reference list and turkey DEGs are given. *p*-values are as determined by Fisher Exact Test with Bonferroni correction.

Among the 204 DEGs shared between lines, 159 were upregulated at 43°C compared to 38°C and 45 down regulated in both lines (denoted in Supplementary Table S2). This identifies a set of and similarly responding genes common to both lines. PANTHER analysis shows significant overrepresentation in these DEGs of genes associated with multiple GO biological process categories including tonic smooth muscle contraction (GO:0014820, 61.18 fold enrichment), regulation of cellular extravasation (GO:0002693, 48.94) regulation of myotube differentiation (GO:0010831, 40.79) sarcomere organization (GO:0045214, 31.58), myofibril assembly (GO:0030239, 22.25) and striated muscle cell development (GO:0055002, 21.85) as well as others.

Ingenuity pathway analysis (IPA, Krämer et al., 2022) was also used to infer biological functions in SCs significantly affected by heat treatment of RBC2 and NCT SCs. Approximately 68% of the turkey genes mapped to IPA identifiers. Canonical pathways were identified for both the RBC2 and NCT temperature comparisons (43°C vs. 38°C) and are summarized in Table 5. Activation z-score represents the change in activation state and thus implicated biological function. Of the pathways with the highest absolute activation z-scores, the majority were signaling pathways with calcium signaling ranking among the highest in both lines.

**TABLE 5:** Significant canonical pathways identified by Ingenuity Pathway Analysis (IPA) for turkey satellite cells following thermal stress. For each line, the 20 pathways with the greatest absolute activation score are given. Pathways are sorted by −log (*p*-value).

Line	Canonical pathways	−log (*p*-value)	z-score	Ratio
RBC2	Dilated Cardiomyopathy Signaling Pathway	7.24	−1.51	0.08
	Calcium Signaling	6.24	1.63	0.06
	Phagosome Formation	4.84	1.53	0.03
	Hepatic Fibrosis Signaling Pathway	4.36	1.60	0.04
	G-Protein Coupled Receptor Signaling	3.76	2.07	0.03
	Opioid Signaling Pathway	3.70	2.83	0.04
	Actin Cytoskeleton Signaling	3.55	2.24	0.04
	CREB Signaling in Neurons	3.14	1.81	0.03
	IL-17 Signaling	3.07	−2.12	0.04
	Synaptogenesis Signaling Pathway	2.69	1.67	0.03
	G Beta Gamma Signaling	2.61	1.34	0.05
	ILK Signaling	2.27	1.63	0.04
	Cardiac Hypertrophy Signaling	2.19	1.34	0.03
	RHOGDI Signaling	2.10	−2.24	0.03
	Erythropoietin Signaling Pathway	1.95	1.63	0.03
	Tumor Microenvironment Pathway	1.93	−2.45	0.03
	SNARE Signaling Pathway	1.84	2.24	0.04
	Signaling by Rho Family GTPases	1.63	1.63	0.03
	eNOS Signaling	1.58	2.00	0.03
	Insulin Secretion Signaling Pathway	0.80	2.00	0.02
NCT	Calcium Signaling	8.65	2.50	0.10
	Phagosome Formation	7.77	3.48	0.06
	G-Protein Coupled Receptor Signaling	7.56	3.16	0.06
	CREB Signaling in Neurons	6.84	3.77	0.06
	Opioid Signaling Pathway	6.68	2.07	0.08
	Breast Cancer Regulation by Stathmin1	4.38	2.04	0.05
	GP6 Signaling Pathway	4.01	2.11	0.09
	Actin Cytoskeleton Signaling	3.53	2.00	0.06
	cAMP-mediated signaling	2.74	2.89	0.06
	Integrin Signaling	2.63	2.53	0.06
	Synaptogenesis Signaling Pathway	2.41	2.67	0.05
	Netrin Signaling	1.71	2.24	0.07
	FAK Signaling	1.36	4.13	0.03
	Superpathway of Inositol Phosphate Compounds	0.41	2.45	0.03
	3-phosphoinositide Degradation	0.41	2.24	0.03
	Necroptosis Signaling Pathway	0.36	2.00	0.03
	D-myo-inositol (1,4,5,6)-Tetrakisphosphate Biosynthesis	0.27	2.00	0.02
	D-myo-inositol (3,4,5,6)-Tetrakisphosphate Biosynthesis	0.27	2.00	0.02
	D-myo-inositol-5-phosphate Metabolism	0.00	2.00	0.02
	3-phosphoinositide Biosynthesis	0.00	2.00	0.02

### Effects of selection (line differences)

Line effects were examined in two pairwise within-temperature comparisons: Control NCT versus RBC2 (38°C) and heat-treated NCT versus RBC2 (43°C) (Table 2). Comparisons between lines at 38°C identified 627 significant DEGs (FDR adjusted *p*-value < 0.05, Table 2) with 159 having |Log_2_FC > 2.0|. Greater gene expression response was observed at 43°C where 7393 DEGs with *p* < 0.05 were identified and 492 of these with |Log_2_FC > 2.0|.

**FIGURE 4:**
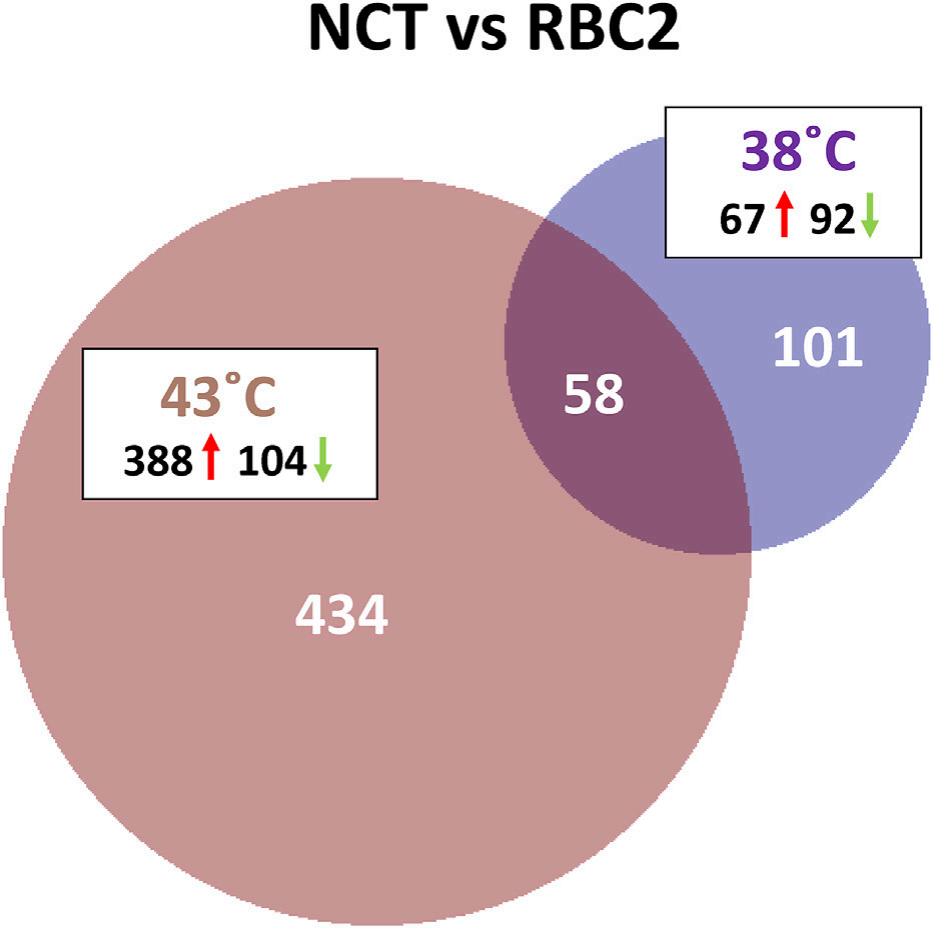
Distribution of differentially expressed genes between lines (F-line versus RBC2) during proliferation of p. major satellite cells. For each temperature comparison, the number of genes with FDR pval <0.05 and |Log_2_FC| > 2.0 that were shared or unique to each incubation temperature are indicated. The number and direction of expression change (↑or ↓) for the genes included in each temperature group are listed outside the Venn diagram. Circle size is proportional to the number of genes.

A total of 593 combined genes were identified with significant DE (*p* < 0.05 and |Log_2_FC| > 2.0) in the comparison between lines. Of these DEGs, 101 were unique to the 38°C comparison, 434 unique at 43°C and 58 shared between the two temperatures (Figure 4). Of the 159 DEGs significant at 38°C, 67 were upregulated and 92 down regulated in the NCT cells compared to RBC2 (Supplementary Table S2).

**FIGURE 5:**
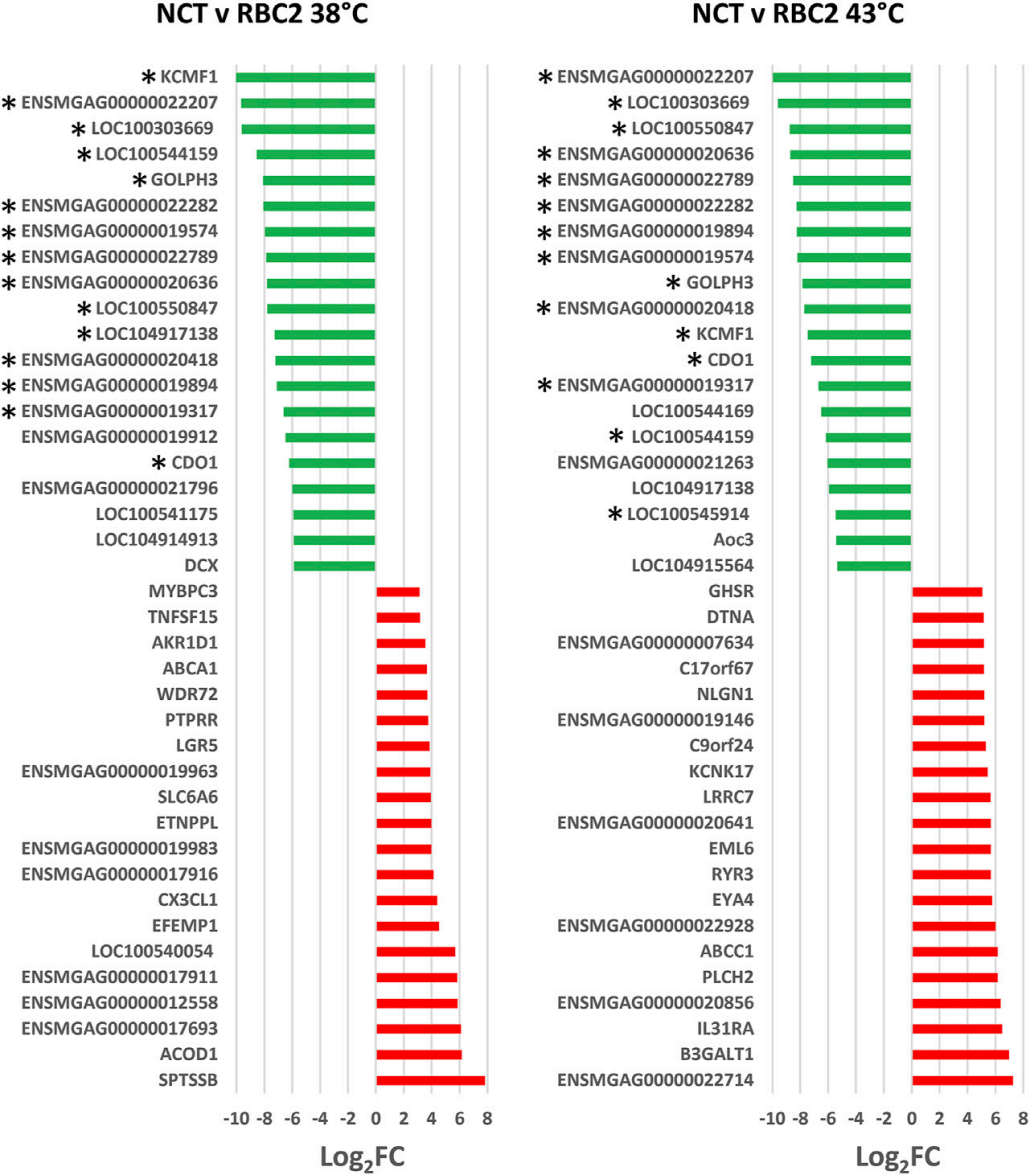
Directional change in the genes showing the highest expression differences at 43°C compared to 38°C. For each with-in line comparison (RBC2 and NCT) the Log_2_FC for each gene is plotted (FDR pval <0.05). Genes shared between temperatures are indicated by asterisks.

The 40 significant DEGs showing the greatest expression differences between the lines are listed in Figure 5. Highly upregulated in NCT cells relative to RBC2 (Log_2_FC = 7.81) was a gene with orthology to *SPTSSB* (serine palmitoyltransferase, small subunit B). In mammals, this enzyme catalyzes the initial rate-limiting step in sphingolipid biosynthesis (Merrill, 1983). Also upregulated were *EFEMP1* (EGF containing fibulin extracellular matrix protein 1) an extracellular matrix glycoproteins of the fibulin family, *LGR5* (leucine rich repeat containing G protein-coupled receptor 5, Log_2_FC = 3.85), a receptor for R-spondins with a potential role in canonical Wnt signaling in the formation and maintenance of adult stem cells, and *PTPRR* (protein tyrosine phosphatase receptor type R, Log_2_FC = 3.75) a member of the protein tyrosine phosphatase (PTP) family that regulate cell growth, and differentiation.

Several ubiquitylation proteins such as *KCMF1* (E3 ubiquitin-protein ligase), *LOC100303669* (ubiquitin-associated protein 2), and *ENSMGAG00000022282* (ubiquitin-conjugating enzyme E2 R2-like), were highly down regulated in NCT compared to RBC2 at 38°C. Ubiquitylation can result in several important enzymatic post-translational modifications. Pathway analysis of the 92 down regulated genes identified the Nicotinic acetylcholine receptor signaling (12.78 fold) and Wnt signaling pathway (8.83 fold) as significantly enriched pathways. Overrepresentation test of the down-regulated genes found highest enrichment for genes involved in cell differentiation and morphogenesis (Table 6).

**TABLE 6:** PANTHER Overrepresentation test of 92 DEGs down regulated in the NCT cells relative to the RBC2 at 38°C after 72 h of proliferation. Shown are the gene ontology categories with the greatest fold enrichment in each category^a^.

	*Gallus gallus* (18109)	Turkey DEGs (52 of 92)	Expected	Fold enrichment	*p*-value
GO biological process complete					
neural crest cell differentiation (GO:0014033)	85	5	0.24	20.49	4.02E−02
mesenchymal cell differentiation (GO:0048762)	152	6	0.44	13.75	4.01E−02
anatomical structure morphogenesis (GO:0009653)	1556	16	4.47	3.58	3.28E−02
GO molecular function complete					
extracellular matrix binding (GO:0050840)	47	4	0.13	29.64	3.18E−02
calcium ion binding (GO:0005509)	598	11	1.72	6.41	2.24E−03
GO cellular component complete					
extracellular region (GO:0005576)	1657	16	4.76	3.36	1.32E−02

a Turkey DEGs were matched to the chicken reference gene list IDs for analysis in PANTHER. For each category, the number of genes in the reference list and turkey DEGs are given. *p*-values are as determined by Fisher Exact Test with Bonferroni correction.

There was a greater number of differentially expressed genes between the lines at 43°C (492 total DEGs) compared to 38°C (Figure 4) and 434 responded uniquely at 43°C (Supplementary Table S2). Expression of 388 genes was significantly higher in the NCT cells with 104 down regulated compared to the RBC2 cells. Among the top up-regulated genes in the NCT line is the ryanodine receptor *RYR3* (Figure 5), one of two sarcoplasmic reticulum calcium channel isoforms (the other being *RYR1*) expressed in skeletal muscle. Both channels are involved in muscle contraction. The RYR3 gene product is a calcium channel that is activated by elevated cytosolic [Ca^2+^] (calcium-induced calcium release) in contrast to RYR1 which is activated by cell depolarization. Of the genes showing the greatest down regulation in the NCT cells at 43°C, 15 were also down regulated at 38°C. Largest fold change was seen for *ENSMGAG00000022207* (a novel protein with BLAST similarity to *HINTWL2*, histidine triad nucleotide binding protein W-like), *LOC100303669* (ubiquitin-associated protein 2), and *LOC100550847* (spindling-like). Spindlin is of interest as it is an activator of Wnt signaling pathway (Su et al., 2014).

Overrepresentation analysis of the 104 down-regulated genes identified two significant GO biological processes; anatomical structure formation involved in morphogenesis (GO:0048646, 6.33-fold enrichment) and multicellular organismal process (GO:0032501, 2.37 fold enrichment). Analysis of the 388 up-regulated genes identified significant enrichment for genes associated with muscle cell differentiation (GO:0042692), muscle structure development (GO:0061061), and vasculature development (GO:0001944) (Table 7). Taken as a whole, NCT cells responded to heat treatment through increased expression of genes involved in muscle development.

**TABLE 7:** PANTHER Overrepresentation test of 388 DEGs up regulated in the NCT cells relative to the RBC2 at 43°C after 72 h of proliferation. Shown are the gene ontology categories with the greatest fold enrichment in each category^a^.

	*Gallus gallus* (18109)	Turkey DEGs (205 of 388)	Expected	Fold enrichment	*p*-value
GO biological process complete					
muscle cell differentiation (GO:0042692)	202	13	2.29	5.69	6.62E−03
muscle structure development (GO:0061061)	332	19	3.76	5.06	1.14E−04
vasculature development (GO:0001944)	352	16	3.98	4.02	2.89E−02
GO molecular function complete					
DNA-binding transcription activator activity (GO:0001216)	311	14	3.52	3.98	4.38E−02
actin binding (GO:0003779)	400	16	4.53	3.53	4.50E−02
cytoskeletal protein binding (GO:0008092)	847	25	9.59	2.61	3.64E−02
GO cellular component complete					
Z disc (GO:0030018)	72	8	0.82	9.82	3.93E−03
I band (GO:0031674)	82	8	0.93	8.62	9.56E−03
sarcomere (GO:0030017)	131	10	1.48	6.74	5.40E−03

a Turkey DEGs were matched to the chicken reference gene list IDs for analysis in PANTHER. For each category, the number of genes in the reference list and turkey DEGs are given. *p*-values are as determined by Fisher Exact Test with Bonferroni correction.

Among the 58 DEGs shared between the temperature treatments, 19 showed higher expression in the NCT cells and 39 showed higher expression in RBC2 at 38°C, identifying a set of genes where expression is similarly changed in both lines regardless of temperature (denoted in Supplementary Table S2). Many of these were among those with expression most highly altered by the heat treatment (Figure 5). In comparison, 56 DEGs show similar expression directionality and only two genes responded directionally different. The first, aldo-keto reductase family 1 member D1 (*AKR1D1*), had significantly higher expression in NCT cells at 38°C (Log_2_FC = 2.4) and lower expression in NCT cells at 43°C (Log_2_FC = −3.8) compared to RBC2 cells. Although function of this gene is mostly associated with catalysis of bile acid intermediates (5-beta-reduction), it also functions in steroid hormone metabolism (Nikolaou et al., 2019). The second gene, doublecortin (*DCX*) had significantly lower expression in NCT cells (Log_2_FC = −5.86) at 38°C and higher expression in at 43°C (Log_2_FC = 2.4) compared to the RBC2 cells. In mammals, DCX has been shown to promote the stability of microtubules (Horesh et al., 1999).

Gene expression differences between the NCT and RBC2 SCs at 43°C were also examined with IPA (11,256 mapped IDs). Here, the Wnt/Ca^2+^ canonical pathway had the highest z-score (analysis cutoffs *p*-value 0.05 and |expression log ratio| > 2.0). Although *Wnt7a* is significantly up regulated in both NCT and RBC2 cells under thermal challenge, *Wnt5a* that signals the Wnt/Ca + pathway was significantly down regulated in NCT versus RBC2. Without restricting log ratio, EIF2 signaling (z = −3.22); members of which were down regulated in NCT cells. As primary targets of several signaling pathways, eIFs regulate gene expression by altering translation in response to stress-related signals.

Direct comparison between the responses of the 1 week SCs in the present study to those of the 7 weeks SCs previously examined (Reed et al., 2017a) is difficult due to the use of different genome annotations for analysis. To facilitate comparison, a composite gene list was created using common gene IDs and the DEGs identified in the two studies (43°C versus 38°C) and these were used to run a Comparison analysis in IPA. Comparison analysis of the RBC2 cells found the majority of the significant canonical pathways to have similar activation (z-score) characteristics. Notable exceptions were Calcium signaling (z = 1.195 and −1.265 in 1 and 7 weeks respectively), EIF2 signaling (z = −0.200 and 2.714 in 1 and 7 weeks respectively) and NRF2-mediated oxidative stress response (z = 0.577 and 2.00 in 1 and 7 weeks respectively). Within the Calcium signaling pathway notable differences included the down regulation of the inositol-triphosphate sarcoplasmic reticulum receptor *ITPR1* [*IP3R*] in both RBC2 cell lines with heat challenge.

**FIGURE 6:**
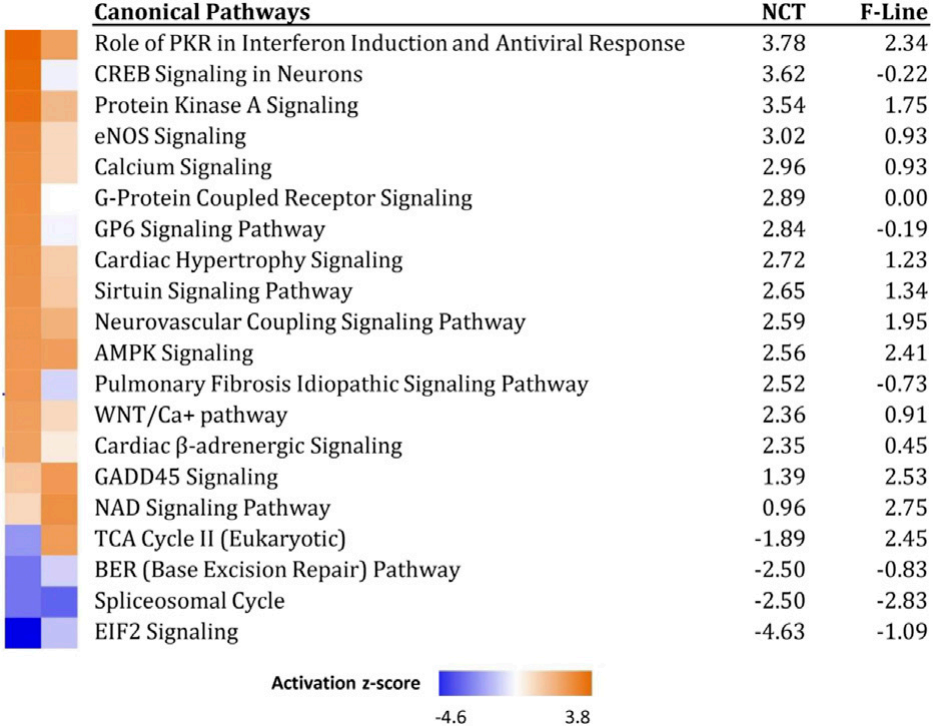
Significant canonical pathways identified in IPA Comparison analysis of genes expressed in NCT versus F-Line turkey skeletal muscle SCs at 43°C. Only the 20 pathways with highest composite Z-scores are shown.

Similar comparison between the responses of NCT SCs to those of SCs from the 16 weeks bodyweight line F (Reed et al., 2017a) was run in IPA. Significant canonical pathways were obtained and the 20 pathways with highest z-score, primarily signaling pathways, are presented in Figure 6. In general, the lines responded similarly within these canonical pathways but with the NCT cells showing often greater z-score activation. Directional changes between the lines are seen for the Pulmonary Fibrosis Idiopathic Signaling and TCA Cycle II pathways. It is unknown whether these differences are due to the age of the birds from which the SCs were isolated (1 versus 7 weeks) or genetic differences resulting from differential selection. Functional differences might be expected between SCs derived from 1 week poults (more actively proliferating SC population) as compared to a more quiescent SC population in 7 weeks birds (Picard and Marcelle, 2013).

## Discussion

Post-hatch thermal stress affects the growth and subsequent structure of poultry breast muscle (Piestun et al., 2017; Patael et al., 2019; Halevy, 2020). Thermal stress has significant effects on the proliferation, differentiation and adipogenic potential of SCs isolated from different genetic lines of turkeys (Clark et al., 2016; Reed et al., 2017a,b) and from commercial birds (Xu et al., 2021a, b). The activation and proliferation of SCs is modulated by signaling molecules which direct myogenesis through signaling pathways. Identification of these pathways provides insight on how selection for growth rate and increased muscle mass may alter the biological response to thermal challenge.

Notch signaling is crucial in the processes by which SCs proliferate, generate differentiating cells, or self-renew. Signaling through HES1 controls transcription of the myogenic transcription factor MYOD1 and the Notch ligand DLL1 thereby determining the activation state of SCs. Although heat stress may alter cell proliferation (Halevy et al., 2001; Xu et al., 2021a), expression of the transcription factor *Pax7*, a marker of proliferation, was similar across treatments. This gene is essential for regulating the expansion and differentiation of SCs (von Maltzahn et al., 2013). Expression of SC proliferation factor *MYOD1* was significantly increased in the 1 week skeletal muscle SCs of both turkey lines with heat stress (Log_2_FC = 2.02 and 1.40 in RBC2 and NCT, respectively). This nuclear protein is a muscle-specific transcriptional regulatory factor. Similar findings for *MYOD1* expression were observed in our previous studies (Reed et al., 2017a; Xu et al., 2022). Like *MYOD1*, significant upregulation in response to heat stress was seen for *DLL1* in both RBC2 and NCT SCs (Log_2_FC = 2.13 and 2.72, respectively). Appropriate oscillation of *DLL1* expression in myogenic cells is important in determining cell fate (Lahmann et al., 2021; Zhang et al., 2021).

Growth processes in the turkey SCs are likely accelerated with heat stress as expression of the differentiation factor myogenin (*MYOG*, Log_2_FC = 5.35 and 3.99 in RBC2 and NC, respectively) was also up regulated. Myogenin is required for differentiation and the formation of multinucleated myotubes. This up-regulation is consistent with the findings of Xu et al. (2022) who measured *MYOG* expression by qRT-PCR. Also consistent with Xu et al. (2022), we observed slightly reduced expression of the peroxisome proliferator-activated receptor *PPARγ* in both lines with heat stress. Expression of myogenic factor 6 (*MYF6* [*MRF4*]), trended slightly lower with heat stress (Log_2_FC = −1.83 and −1.37 in RBC2 and NCT, respectively). Recent studies in mice found this myogenic regulator checks muscle stem cell exhaustion by transcriptionally regulating multiple myokines and blocking premature differentiation (Lazure et al., 2020).

Other signaling molecules importantly modulate activation of SCs. Three fibroblast growth factors (FGFs), a family of proteins important in cell signaling for normal cellular development, showed significant expression differences. Notably, *FGF12* was upregulated in the NCT cells at 43°C compared to control (38°C) and *FGF10* was significantly upregulated in the NCT cells relative to the RBC2 at 43°C (Log_2_FC = 2.02). A third FGF (*FGF19*) was down regulated by heat stress in the RBC2 cells (43 versus 38°C) and was lower in NCT cells compared to RBC2 in controls (38°C). Although specific function has not been assigned, in mammals FGF10 binds to fibroblast growth factor receptors (FGFRs) but FGF12 does not. Expression of *FGF19* in humans is reported in the ileum in response to bile acid absorption (Potthoff et al., 2012). The cytokine *LIF* (leukemia inhibitory factor) involved in the induction of differentiation, was significantly down regulated in both the RBC2 and NCT cells (Log_2_FC = −2.60 and −1.83, respectively) with heat stress.

Previous transcriptome analysis of SCs from 7-week old birds (Reed et al., 2017a) found thermal stress alters expression of several Wnt genes. In the present study of SCs from 1-week old birds, *Wnt7a* was the most highly upregulated gene in the RBC2 line (Log_2_FC = 7.94) and was significantly upregulated in NCT SCs (Log_2_FC = 3.37) under heat stress. In the mouse, the Wnt/PCP pathway is regulated through the frizzled-7 (Fzd7) receptor and stimulates SC proliferation, promotes hypertrophy of myofibers resulting in increased skeletal muscle mass (Otto et al., 2008; Le Grand et al., 2009). Up regulation of *Wnt7a* during the period of SC peak mitotic activity, suggests that thermal stress affects turkey skeletal muscle through a similar mechanism. Supporting evidence is provided by knockdown experiments where in the same cultured 1-week turkey SCs used in this study, knockdown of *Fzd7* significantly decreased proliferation and *MYOD* expression especially under heat stress (Xu et al., 2022). The NCT SCs being less affected than RBC2 SCs. Results from these combined studies show that growth selection appears to have altered the response to thermal stress through the Wnt/PCP pathway affecting poultry breast muscle growth (myofiber hypertrophy), structure, and protein to fat ratio (fat deposition).

As a ubiquitous intracellular signal, Ca^2+^ is vital in regulation of skeletal muscle development, maintenance and regulation through calcium signaling. Calcium regulation in SCs appears to be largely affected by heat stress and the Calcium signaling pathway ranked among the highest in IPA by z-score. The spatiotemporal pattern of Ca^2+^ signaling in developing muscle is dependent on stores of Ca^2+^ (Tu et al., 2016). Expression of the two major calcium channels inositol-triphosphate receptors (IP3R) and RYR (ryanodine receptors) are developmentally regulated. Expression of the *IP3* receptor was not significantly altered by heat stress in the turkey SCs, but *RYR3* was among the most highly upregulated genes. RYR3 is a peripheral calcium channel associated with the sarcoplasmic reticulum (SR). In mice, *RYR3* has a distinct developmental expression profile with maximum expression in neonates followed by decline, most noticeable in fast-twitch muscles (Tarroni et al., 1997). Unlike RYR1, which is coupled to the voltage-gated calcium channel and is activated by depolarization, RYR3 is only susceptible to calcium-induced Ca release (CICR, not voltage-gated). Expression of the SR luminal calcium-binding protein calsequestrin (*CASQ2*) is also elevated. CASQ is responsible for storage of Ca^2+^ within the SR when the muscle is at rest, and appears to be involved in regulation of RYR1 and possibly RYR3 calcium release activity. Studies in turkeys suggest that splice variants of ryanodine receptors are altered by heat stress, which could affect postmortem calcium homeostasis and meat quality (Strasburg and Chiang, 2009).

Perez et al. (2005) used mouse dyspedic myotubes lacking genes for all ryanodine receptors (RYR1, RYR2, and RYR3) to investigate the effects of differential expression of *RYR1* and *RYR3*. They showed that increasing expression of *RYR3* resulted in increased cytosolic [Ca^2+^]. Because increased temperatures result in increased expression of *RYR3*, it is likely that will result in increased resting cytosolic calcium levels and increased gene transcription. Predicted upregulation of plasma membrane voltage-gated calcium channel would also likely increase cytosolic calcium via increased channel abundance although compensatory mechanisms may control the flow of Ca^2+^ through the channels. Also, a potential effector is CICR, releasing ions when Ca^2+^ levels reach a concentration threshold. Higher cytosolic Ca^2+^ levels activate calmodulin with downstream effects resulting in enhanced transcription leading to cell growth and development, consistent with the increased growth of the SC cells. The trends in the calcium signaling pathway were similar among the RBC2 and NCT lines but the magnitude of fold changes was greater in the commercially selected NCT birds. Modulation of calcium-regulating genes in early development could potentially be associated with aberrant postmortem Ca^2+^ regulation tied to meat quality defects in heat-stressed birds (Sporer et al., 2012).

Response to heat stress, particularly in NCT cells is reflected in gene expression supporting early myogenesis. Pathway analysis also highlighted increased in expression in myofibrillar proteins (e.g., troponin T, alpha-actin). Most of these genes appear to be of the fast muscle isoforms. Expression of the short transient receptor potential channel 3 (*TRPC3*) was down regulated (but only in the NCT cells). Ca^2+^ signaling through calcineurin via TRPCs has downstream phosphorylation of Ca^2+^-sensitive transcription factors (*NFAT*, nuclear factor of activated T cells) (Tu et al., 2016). Heat-stressed NCT SCs highly upregulated *NFATC2* compared to control (Log_2_FC = 5.79) and expression was significantly greater in NCT cells compared to RBC2 cells at 43°C. In mice, deficiency of NFATC2 results in impaired myoblast fusion (Horsley et al., 2001). Expression of *NFATC3* was also significantly higher in NCT SCs at 43°C (Log_2_FC = 2.07). Knockout of *NFATC3* in early myogenesis results in reduced muscle mass (Kegley et al., 2001). Differential response of these transcription factors may be the result of selection for muscle growth and confirmation characteristics in commercial birds.

## Conclusions

This study has identified genes and gene pathways in proliferating 1 week turkey SCs altered by phenotypic selection and heat stress. This RNAseq analysis provides a snapshot, along a continuum of gene expression changes that may translate into functional differences in gene products. Although these SCs are still proliferating, and are not yet differentiated into muscle fibers, this study provides clues into how SC activity, cellular fate, and ultimately muscle development are altered by heat stress and highlight cellular processes for future study. Heat-treated SCs respond with significant expression changes in several key signaling pathways and muscle-related structural genes. In general, SCs responded by upregulating muscle components with differential response from growth-selected birds in key signaling pathways including Wnt and calcium signaling. We hypothesize that heat stress causes higher resting cytosolic calcium levels in part due to changes in RYR3 and IP3R expression. Further studies will examine the downstream effects of these gene expression changes on cytosolic calcium in SCs and skeletal muscle of developing poults.

## Data Availability

The datasets presented in this study can be found in online repositories. The names of the repository/repositories and accession number(s) can be found below: https://www.ncbi.nlm.nih.gov/genbank/, PRJNA842679.
